# Graphene Oxide–Silver Nanocomposite Enhances Cytotoxic and Apoptotic Potential of Salinomycin in Human Ovarian Cancer Stem Cells (OvCSCs): A Novel Approach for Cancer Therapy

**DOI:** 10.3390/ijms19030710

**Published:** 2018-03-01

**Authors:** Yun-Jung Choi, Sangiliyandi Gurunathan, Jin-Hoi Kim

**Affiliations:** Department of Stem Cell and Regenerative Biotechnology, Konkuk University, Seoul 05029, Korea; choi_yunjung@naver.com

**Keywords:** reduced graphene oxide–silver nanocomposite (rGO–Ag), human ovarian cancer cells, ovarian cancer stem cells (OvCSCs), cytotoxicity, apoptosis

## Abstract

The use of graphene to target and eliminate cancer stem cells (CSCs) is an alternative approach to conventional chemotherapy. We show the biomolecule-mediated synthesis of reduced graphene oxide–silver nanoparticle nanocomposites (rGO–Ag) using R-phycoerythrin (RPE); the resulting RPE–rGO–Ag was evaluated in human ovarian cancer cells and ovarian cancer stem cells (OvCSCs). The synthesized RPE–rGO–Ag nanocomposite (referred to as rGO–Ag) was characterized using various analytical techniques. rGO–Ag showed significant toxicity towards both ovarian cancer cells and OvCSCs. After 3 weeks of incubating OvCSCs with rGO–Ag, the number of A2780 and ALDH^+^CD133^+^ colonies was significantly reduced. rGO–Ag was toxic to OvCSCs and reduced cell viability by mediating the generation of reactive oxygen species, leakage of lactate dehydrogenase, reduced mitochondrial membrane potential, and enhanced expression of apoptotic genes, leading to mitochondrial dysfunction and possibly triggering apoptosis. rGO–Ag showed significant cytotoxic potential towards highly tumorigenic ALDH^+^CD133^+^ cells. The combination of rGO–Ag and salinomycin induced 5-fold higher levels of apoptosis than each treatment alone. A combination of rGO–Ag and salinomycin at very low concentrations may be suitable for selectively killing OvCSCs and sensitizing tumor cells. rGO–Ag may be a novel nano-therapeutic molecule for specific targeting of highly tumorigenic ALDH^+^CD133^+^ cells and eliminating CSCs. This study highlights the potential for targeted therapy of tumor-initiating cells.

## 1. Introduction

Ovarian cancer is the sixth most common malignancy and fifth most common disease in women worldwide. More than 200,000 new cases are diagnosed each year worldwide, accounting for 4% of all cancers [1,2]. Unfortunately, most cases are diagnosed in advanced stages or when the disease has metastasized in the ovaries [3]. Furthermore, a high degree of heterogeneity within ovarian tumors between different ovarian cancer subtypes is a key feature of the disease, and the lack of widely expressed or therapeutically targetable genetic changes restricts effective treatment options [4]. Regardless of advances in treatment, epithelial ovarian cancer is considered one of the most lethal gynecologic malignancies. The standard therapy management generally involves a combination of surgical tumor debulking and chemotherapy [5]. Over the past few decades, combination therapy and chemotherapy have been the standard treatments and involve a combination of intravenous platinum and taxane chemotherapy for advanced cancer [6]. Although numerous molecular targeting agents are available, the standard combination of surgery and chemotherapy for treating ovarian cancer results in recurrence in 70% of patients who undergo the first-line treatment within 18 months [1,7]. Several recent studies reported that cancer stem cells (CSCs) are involved in drug resistance and cancer recurrence [8]. Ovarian cancer cells comprise a heterogeneous population of cells with distinct properties and functions. Some of these cells exhibit increased tumorigenicity and differentiating capacity and are called CSCs [9,10].

CSCs are typically isolated and identified based on either differential expression of cell surface markers or differential biochemical properties [11,12,13]. Aldehyde dehydrogenase (ALDH) together with CD133 serve as markers to identify CSC populations in hepatocellular carcinoma [14]. In ovarian cancer, ALDH^+^ cells are present in most tumors and are capable of directly generating tumors in vivo [13]. Kryczek et al. [12] demonstrated that ALDH and CD133 expression could be partially rescued under in vitro serum-free and sphere culture conditions and by in vivo passage in immune-deficient xenografts, but the expression of CD24, CD44, and CD117 could not be recovered in such a manner. Because of the high expression levels of stem cell core gene transcripts, ALDH^+^ and CD133^+^ cell populations formed three-dimensional spheres more efficiently than their negative counterparts. Among four different subpopulation of cells, ALDH^+^CD133^+^ cells could generate all four ALDH^+/−^CD133^+/−^ cell populations and larger tumors more rapidly than their negative counterparts [12,15]. Although chemotherapy is one of the most effective strategies for treating malignant tumors, patient relapse still occurs. Further, metastasis of malignant cells is very common and has severe side effects. Therefore, developing an alternative treatment approach using biocompatible, biodegradable, and self-regulating nanomaterials in vitro and in vivo is essential [16]. Nanotechnology has the potential to overcome current chemotherapeutic barriers in cancer treatment because of its unique physical, chemical, and biological properties. Particularly, graphene has gained attention for nanotherapy.

Graphene oxide (GO) consists of oxidized sheets of graphite oxide, in which the basal planes, decorated mostly with epoxide and the hydroxyl groups, contain only one or few layers of carbon atoms, such as graphene, which can be reduced to graphene-like sheets by removing the oxygen-containing groups with the recovery of a conjugated structure [17,18,19]. GO is used as a precursor material to synthesize graphene. Graphene is a two-dimensional sp^2^-bonded carbon material with a honeycomb crystal lattice structure. Graphene has potential applications in engineering, electronics, medicine, energy, industrial, and household design appliances [20,21,22,23]. Graphene has been used for several biomedical applications because of its excellent mechanical, electrical, thermal, optical, elastic, and biological properties. Therefore, the production of high-quality graphene is necessary.

High quality of graphene is synthesized using a variety of methods including chemical vapor deposition; however, the produced graphene is unsuitable for mass productions [24,25]. Several physical and chemical methods have been developed to reduce GO. The reduced GO (rGO) sheets can be prepared by chemical and mechanical exfoliation, epitaxial growth [26], chemical vapor deposition [27], and chemical reduction [19]. Although GO reduction is important, the final product is difficult to obtain, and different reduction processes produce different properties, in turn affecting the final performance of materials or devices composed of rGO [18]. The most conventional chemical method seems to be feasible but toxic, which is due to usage of a variety of chemical reducing agents and also there are limitations such as solubility, irreversible agglomeration, and toxicity to living organisms [19,22]. Therefore, alternative methods such as biological methods that are environmentally friendly and biocompatible must be developed.

The synthesis of composites containing graphene with silver (i.e., grapheme–silver nanocomposite) has been explored for their properties and applications. Silver nanoparticles (AgNPs) have attracted much attention because of their antibacterial, antifungal, antiviral, and anti-cancer properties [28,29]. AgNPs have been used for surface-enhanced Raman scattering because their particle size and shape can be regulated [30]. Recently, several studies have reported the synthesis of graphene–silver nanocomposites using green methods, such as microwave irradiation [31] and methods involving using biomolecules such as gelatin [32], bacteria [33], tryptophan [34], and plant extracts [35].

Nanocomposites can be prepared by simple processing with lower loading than conventional polymer composites and have lower component weights. Moreover, the multifunctional property enhancements made possible with nanocomposites may allow for new applications of polymers [36]. Presently, graphene composites with various metal NPs have been used as antibacterial agents [31], optoelectronics, super capacitors [37], and anti-cancer agents [35]. To overcome aggregation, the use of surfactants as stabilizing agent molecules is necessary [38]. Therefore, the use of a novel biomolecule to produce AgNPs–rGO films is necessary. In this study, we used R-phycoerythrin as a reducing and stabilizing agent to synthesize rGO–Ag nanocomposites. Phycoerythrin is a major light-harvesting pigment and phycobiliprotein of red algae. R-phycoerythrin (RPE) is commonly used as a fluorescent label [39]. Phycobiliproteins are known for their immuno-enhancing, anti-inflammatory, anti-carcinogenic, and antioxidant nutritive effects and anticancer properties [40].

Although numerous studies have evaluated the effect of nanoparticles on several cancer cell lines, the role of rGO–Ag in ovarian cancer stem cells has not been investigated. Therefore, we evaluated the apoptotic efficiency of rGO–Ag in ovarian cancer cells and different subpopulations of ovarian cancer stem cells. The first objective was to synthesize silver nanoparticles, graphene oxide, reduced graphene oxide, and rGO–Ag using R-phycoerythrin. The second objective was to evaluate the cytotoxic potential of silver nanoparticles, graphene oxide, reduced graphene oxide, and rGO–Ag in ovarian cancer cells and ovarian cancer stem cells (OvCSCs). The third objective was to investigate the mechanisms of toxicity of rGO–Ag in OvCSCs. The final objective was to evaluate the effect of rGO–Ag and salinomycin and the combination effect of rGO–Ag and salinomycin on cytotoxicity of OvCSCs.

## 2. Results and Discussion

### 2.1. Synthesis and Characterization of GO, rGO, rGO–Ag and AgNPs

**Figure 1:**
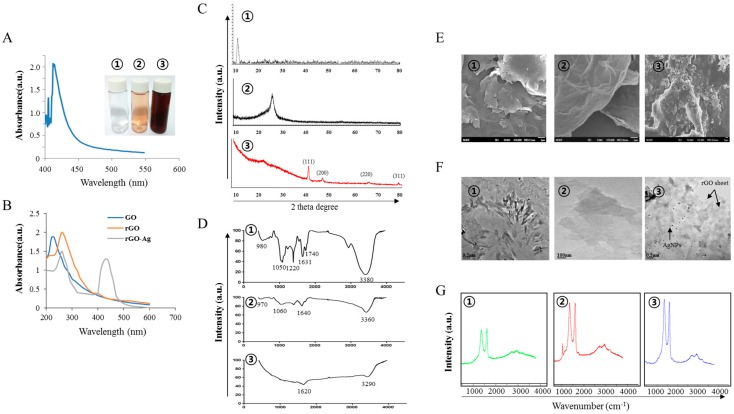
Synthesis and characterization of graphene oxide (GO), reduced graphene oxide (rGO), and rGO–Ag. (**A**) Synthesis of AgNPs using RPE. AgNPs exhibited a maximum absorption peak at ~430 nm corresponding to the surface plasmons and presence of AgNPs. The inset shows the tubes containing silver nitrate (1) RPE (2) silver nitrate and RPE (3); (**B**) Spectra of GO showed a maximum absorption peak at ~230 nm corresponding to the π–π transitions of aromatic C–C bonds. The inset shows color of GO (1), rGO (2), and rGO–Ag (3); (**C**) X-ray diffraction (XRD)pattern of GO (1), rGO (2), and rGO–Ag (3); (**D**) Fourier-transform infrared spectroscopy (FTIR) spectra of GO (1), rGO (2), and rGO–Ag (3); (**E**) SEM images of GO (1), rGO (2), and rGO–Ag (3); (**F**) Transmission electron microscopy (TEM) images of GO (1), rGO (2), and rGO–Ag (3); (**G**) Raman spectroscopy of GO (1), rGO (2), and rGO–Ag (3).

To synthesize the rGO–Ag, we prepared essential precursors such as AgNPs, GO, and rGO. First, extracellular synthesis of AgNPs was carried out using RPE with 5 mM AgNO_3_ aqueous solution. RPE is a fluorescent phycobiliprotein [41]. Reduction of the silver ion in AgNPs was observed as a color change from the original pinkish color, a characteristic color of RPE, to dark brown (Figure 1A inset). Figure 1A shows the UV–Vis spectrum of synthesized AgNPs by the characteristic features of a dark brown color [42,43]. Maximum absorbance was observed at approximately 430 nm. Mahdieha et al. [44] demonstrated the synthesis of AgNPs using *Spirulina platensis*, a blue-green micro algae (cyanobacteria) known to contain phycobiliproteins. Similarly, another phycobili protein known as C-phycocyanin was used to biosynthesize AgNPs [45]. Patel et al. [45] observed that C-phycocyanin incubation with AgNO_3_ lost its characteristic absorbance at 620 nm after 12 h, suggesting that the pigment was denatured by AgNO_3_. Bekasova et al. [46] demonstrated the synthesis of AgNPs using RPE extracted from the red algae *Callithamnion rubosum.* Our data are consistent with those of previous studies suggesting that phycobiliproteins reduce AgNO_3_. These experiments suggest that protein-based pigment from cyanobacteria mediates the formation of nanoparticles [44,45] through the presence of amino acids, vitamins, and carbohydrates. We explored the possibility of using RPE to reduce graphene oxide and synthesize rGO–Ag. To produce rGO–Ag, graphene oxide (GO) was prepared via the modified Hummer method [47] by oxidizing graphite. The synthesized GO exhibits two characteristic peaks at 230 and 300 nm, corresponding to the π–π* transitions of aromatic C–C bonds and *n*–π* transitions of C=O bonds, respectively, whereas rGO exhibited a band at 263 nm, indicating restoration of the extensive conjugated sp^2^ carbon network [21,22,48]. (Figure 1B). Figure 1B inset shows the color of GO, rGO, and rGO–Ag. The GO dispersion was obtained by the oxidation of graphite; the resulting solution was clear and a homogeneous yellow-brown GO dispersion. After the reduction of GO by RPE, the color changed from pale-yellow to black, indicating the reduction of GO. The aqueous dispersions of GO and resulting rGO showed a distinct color change after chemical reduction. Such observations support the formation of rGO. The combination of the graphene–silver for well-dispersed rGO–Ag was visibly observed as a distinct color change from black to dark brown [35] (Figure 1B inset). For rGO–Ag nanocomposites, absorption signals were observed for both Ag and rGO (Figure 1B)**,** and the presence of AgNP and rGO peaks within the composite clearly indicated the synthesis rGO–Ag.

The structural properties of the GO, rGO, and rGO–Ag samples were characterized by X-ray diffraction (XRD). The XRD pattern of GO exhibited a strong peak at 2*θ* = 11.7°, corresponding to an interlayer spacing of approximately 0.76 nm, indicating the presence of oxygen functionalities that facilitated the hydration and exfoliation of GO sheets in aqueous media [22,49]. The characteristic peak of graphite at 26.58° disappeared after oxidation, while an additional peak at 11.7° was observed (Figure 1C), corresponding to the diffraction peak of GO [50]. The broad peak centered at 2*θ* = 25.8° in the XRD pattern of the rGO sample confirmed random packing of the graphene sheets in rGO [35]. Interestingly, the XRD patterns of rGO–Ag showed characteristic peaks at 2*θ* = 33.42°, which were related to the (111) planes of face-centered cubic of Ag, suggesting successful synthesis of Ag nanoparticles on rGO. The results are consistent with the properties of rGO–Ag produced by various other methods including microwave-assisted green synthesis of Ag/reduced graphene oxide [51], the solvothermal method [52], and plant extracts [35].

FTIR was performed to confirm the reduction of GO by RPE. The GO sheet showed apparent adsorption bands at 980 cm^−1^ (for vibrations from epoxy, ether, or peroxide groups), alkoxy C–O (1050 cm^−1^), epoxy C–O (1220 cm^−1^), aromatic C=C (1631 cm^−1^), carboxyl C=O (1740 cm^−1^), and hydroxy –OH (3380 cm^−1^) groups (Figure 1D). The presence of oxygen-containing functional groups, such as C=O and C–O, suggested that the graphite was oxidized into GO, which is consistent with the results of previous studies [21,53]. In the FTIR spectra of rGO, the presence of a broad band at 3360 cm^−1^ (for O–H stretching vibrations), intense broad bands at 1640, and weak band 1060 cm^−1^ (for C–O breathing vibrations) and 970 cm^−1^ (for vibrations from epoxy, ether, or peroxide groups) indicated the reduction of oxygen functional groups in the GO structure [21,53]. After RPE reduction, the intensity of bands associated with oxygen functional groups was greatly decreased, indicating the removal of oxygen functional groups on rGO. As shown in Figure 1, the functional group GO was significantly reduced in the rGO–Ag, e.g., C=O carbonyl stretching (1620 cm^−1^) and hydroxy–OH (3290 cm^−1^) were decreased [35,54,55].

To determine the surface morphology of GO, rGO, and rGO–Ag, we performed scanning electron microscopy (SEM) analysis. As shown in Figure 1E, GO was observed as single flakes, and its morphology resembled a thin curtain, with silky closely packed lamellar and assembled paper-like materials [22,56,57]. rGO showed a large surface with sharp edges and compact structure. The morphology of rGO exhibited curvy, wrinkled, and paper-like sheet morphology. Chemically reduced GO showed agglomeration of exfoliated platelets [58]. The typical reduced GO showed well-exfoliated but aggregated and crumpled silk waves and appeared as flat stacked rGO sheets [59]. The rGO mostly consisted of single- and few-layer sheets. During the reduction process, rGO was partially repaired from sp^3^ hybridized carbon atoms and the number of the sp^2^ domains was increased, while the sizes of the sp^2^ domains decreased [19,60]. This suggests the presence of at least 2–3 layers of reduced GO sheets, as the reported thickness for the single-layer reduced GO sheet is ~1 nm [61]. RGO-based gels have a large amount of steric space [62]. SEM images of the graphene film after modification with AgNPs are shown in Figure 1E. Biological molecule-mediated functionalization provided separation of individual GO sheets, which was comparable to the chemical functionalization of GO [63] and both larger and smaller Ag particles coexisted on the rGO sheet. In support of our results, Jiao et al. [62] observation similar structural arrangements of RGO/silver nanoparticle composite hydrogels by the co-reduction of silver ions and GO in the presence of vitamin C.

rGO–Ag was obtained after co-reduction of silver ions and GO to form rGO–Ag in the presence of RPE. Transmission electron microscopy (TEM) images of GO revealed a closely packed lamellar and plate structure with a clean surface (Figure 1F). rGO sheets appeared to be stacked in irregular layers with few wrinkles and little folding and were entangled with each other. Further, TEM micrographs of the rGO sheets clearly showed the lattice borders of graphene. As shown in Figure 1F, AgNPs were homogeneously deposited on rGO sheets with uniform sizes [35,64]. It was clearly demonstrated that Ag nanoparticles were anchored around the surface of the rGO; the presence of RPE catalyzed the reduction of AgNO_3_ to Ag, resulting in the reduction of AgNO_3_ on the rGO surface.

Raman Spectroscopy is a widely used technique for characterizing carbon products and can reveal the crystal structure of carbonaceous materials and can distinguish the order, disorder, and defects in carbon structures. Raman intensities can also be measured in conjugated and double carbon-carbon bonds [65,66,67]. Here, we examined the electronic and structural properties of GO, rGO, and rGO–Ag. As shown in Figure 1G, in the Raman spectrum of GO, the D and G bands were located at 1343 and 1604 cm^−1^, respectively. The D band was assigned to the breathing mode of the K-point phonons with A_1g_ symmetry, whereas the G band introduced the E_2g_ phonon of carbon sp^2^ atoms [19,22,68]. The Raman Spectrum of rGO showed the D band at 1347 cm^−1^ and G band at 1607 cm^−1^ (Figure 1G). The ratio of I_D_/I_G_ increased to 1.805 (rGO) from 1.64 (GO) [22]. The relative intensity of the two main peaks such as D and G of the Raman spectra indicated the efficiency of reduction of GO by the reducing agent [69]. In the Raman spectra of rGO–Ag, the G bands were broadened and the D bands were intensified, which is due to enhanced disorder of the rGO and rGO–Ag [35]. The highest intensity ratio of rGO indicated disorder on the graphene sheets after reduction and an increased number of sp^2^ domains. These results suggest that the reduction of GO caused fragmentation and yielded smaller rGO graphitic domains with different sizes or recovered graphitic electronic conjugation for rGO. In addition, the rGO–Ag may have been more defective and disordered at active sites for the adsorption of other molecules [70,71,72,73].

### 2.2. Effect Of GO, rGO, rGO–Ag, and AgNPs on Ovarian Cancer Cells

**Figure 2:**
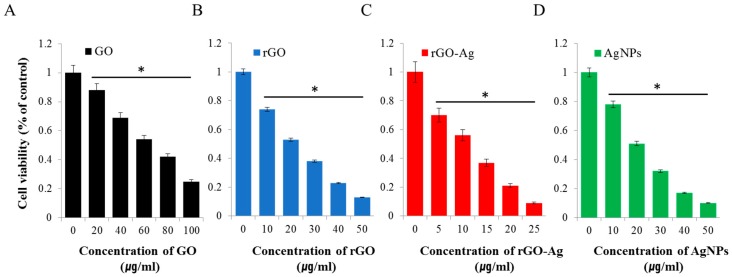
Effects of GO, rGO, rGO–Ag, and AgNPs on cell viability of human ovarian cancer cells. The viability of A2780 human ovarian cancer cells was determined after 24-h exposure to different concentrations of GO (**A**), rGO (**B**), rGO–Ag (**C**), and AgNPs (**D**) using the CCK-8 assay. The results are expressed as the mean ± standard deviation of three independent experiments. The viability of treated cells compared to untreated cells was analyzed using Student’s *t*-test (* *p* < 0.05).

To determine the effect of four different nanomaterials on ovarian cancer stem cells, we first examined the effect of all the prepared nanomaterials on (bulk) parental cells, ovarian cancer cells (A2780). To assess the efficiency of the prepared rGO–Ag, the cells were incubated with the rGO–Ag, including other control samples such as GO, rGO, and AgNPs for 24 h. As shown in Figure 2, dose-dependent inhibition of cell viability was observed depending on nanomaterials. For example, GO, rGO, rGO–Ag and AgNPs had respective IC_50_ values of ~60, 20, 2 and 20 µg/mL (Figure 2A–D). Among the tested materials, the rGO–Ag was shown to have a more pronounced inhibitory effect on cell viability compared to the other tested nanomaterials. Interestingly, this rGO–Ag was highly effective and more cytotoxic at lower concentrations than the other tested nanomaterials because of the anchoring of smaller size silver nanoparticles with an average size of 10 nm on the surface of the graphene sheets [35]. Overall, these results suggest that the rGO–Ag is a promising material for inhibiting the cell viability of ovarian cancer cells and ovarian cancer stem cells.

Several studies reported that GO is less toxic than rGO in various types of cancer cells because of the functionalization of different types of reducing agents used for reduction and the oxidation efficiency of GO [22,28,74,75]. Interestingly, the combination of graphene and silver showed a more pronounced effect. For example, the anticancer activity of rGO sheets resulted from reduction by glucose in the presence of a Fe catalyst [76]. Similarly, rGO–AgNP–folic acid showed significant solubility and toxicity against HeLa cells [77]. Recently, Fiorillo et al. [78] observed a dose-dependent and selective inhibitory effect of tumor sphere formation in the presence large GO flakes in CSCs of ovarian, prostate, pancreatic, and lung cancers as well as glioblastoma [78]. Our cell viability assay results suggest that the obtained rGO–Ag had a stronger inhibitory effect than GO, rGO, and AgNPs. In addition, each tested nanomaterial had a distinct cell viability profile. Based on our results and previously published data, the rGO–Ag was more cytotoxic in cancer cells [35]. It was observed that the rGO–Ag had IC_50_ values that were at least several fold lower compared to the other nanomaterials tested. Generally, the inhibitory action of nanomaterials in OvCSCs differed from that in bulk cancer cells. Therefore, we examined whether the rGO–Ag can efficiently induce cell death of OvCSCs. To compare the efficiency of tested nanomaterials with OvCSCs, we used IC_50_ concentrations of 60, 20, 2, and 20 µg/mL for GO, rGO, AgNPs, and rGO–Ag, respectively, which were optimized in parental cells.

### 2.3. Isolation and Characterization of OvCSCs

**Figure 3:**
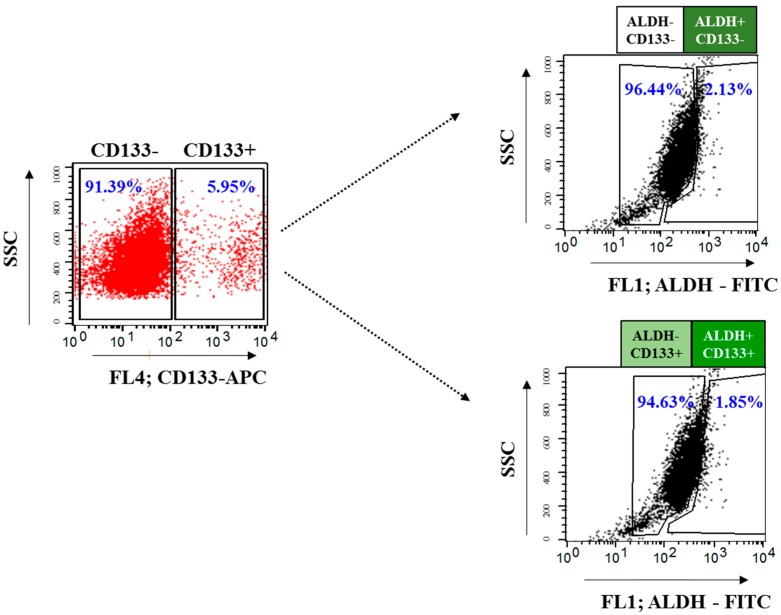
Schematic representation of expression of CSC markers of ALDH^+^ and CD133^+^ in human ovarian cancer cells using FACS.

To determine the cytotoxic potential of GO, rGO, rGO–Ag, and AgNPs in different OvCSCs subpopulations, we first gated CD133 expression and then examined the expression of ALDH in the CD133^−^ and CD133^+^ cell populations, and the tumorigenic potential of different subpopulations of cells was determined using ALDH expression and ALDH activity in four different subpopoulation of cells including ALDH^+^CD133^+^, ALDH^−^CD133^+^, ALDH^+^CD133^−^, and ALDH^−^CD133^−^ cells [11,13,15,79,80,81]. Silva et al. [13] reported that ALDH was the only potential stem cell marker expressed in all primary tumor specimens and was detected in limited cellular sub-populations of human primary tumor cells (Figure 3). Thus, ALDH is a potentially useful CSC marker in ovarian cancer. Huang et al. [82] found that ALDH^+^/CD133^+^ cells increased the generation of tumor xenografts when ALDH and CD133 were used together compared to using ALDH^+^/CD133^–^ or ALDH^+^ alone [82]. ALDH^+^/CD133^+^ cells tended to have larger tumors which were stimulated more rapidly than ALDH^+^/CD133^–^ cells [82,83].

### 2.4. Effect of rGO–Ag on Cell Viability of OvCSCs

**Figure 4:**
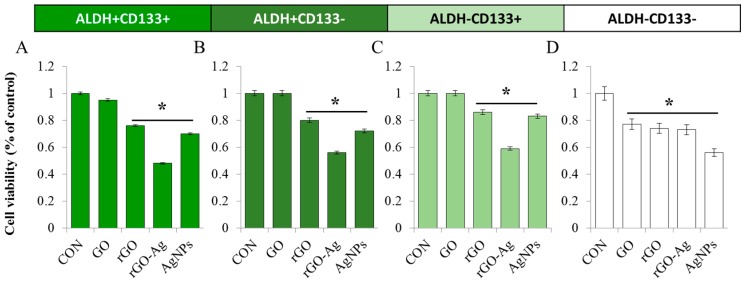
Effects of GO, rGO, rGO–Ag, and AgNPs on cell viability of various subpopulations of OvCSCs. The viability of ALDH^+^CD133^+^ (**A**), ALDH^−^CD133^+^ (**B**), ALDH^+^CD133^−^ (**C**), and ALDH^−^CD133^−^ (**D**) cells was determined after 24-h exposure to GO (60 µg/mL), rGO (20 µg/mL), rGO–Ag (2 µg/mL), and AgNPs (20 µg/mL) using the CCK-8 assay. The results are expressed as the mean ± standard deviation of three independent experiments. The viability of treated cells compared to untreated cells was analyzed using Student’s *t*-test (* *p* < 0.05).

Previous studies suggested that GO exerts its effects on CSCs by inhibiting several key signal transduction pathways, but it is not toxic to bulk cancer cells [78]. However, no studies have examined the differential cytotoxicity of the rGO–Ag in OvCSCs. Therefore, we explored the possibility of identifying effective nanomaterials for cancer stem cells in different subpopulations of cells, which are known to be metastasis-initiating cells. To address this issue, we treated different subpopulations of OvCSCs, including ALDH^+^CD133^+^, ALDH^+^CD133^−^, ALDH^−^CD133^+^, and ALDH^−^CD133^−^, isolated from ovarian cancer cell lines with GO (60 µg/mL), rGO (20 µg/mL), rGO–Ag (2 µg/mL), and AgNPs (20 µg/mL) for 24 h (Figure 4A–D). All four types of OvCSCs were treated with the IC_50_ value of each nanomaterial; the results of the cell viability assay suggested that the inhibitory ability of the rGO–Ag was considerably greater compared to that of the other tested nanomaterials. Interestingly, a more suppressive effect was observed in ALDH^+^CD133^+^ compared to other subpopulations. This finding is clearly consistent with our results, demonstrating the toxicity of AgNPs against various subpopulations of OvCSCs. Overall, we found that ALDH^+^/CD133^+^ cells were more sensitive with respect to rGO–Ag, and rGO–Ag appeared to significantly eliminate CSCs molecules relative to the tumorigenic potential population of ALDH^+^/CD133^+^ cells [15].

### 2.5. Effect of rGO–Ag Determined by Clonogenic Assay

**Figure 5:**
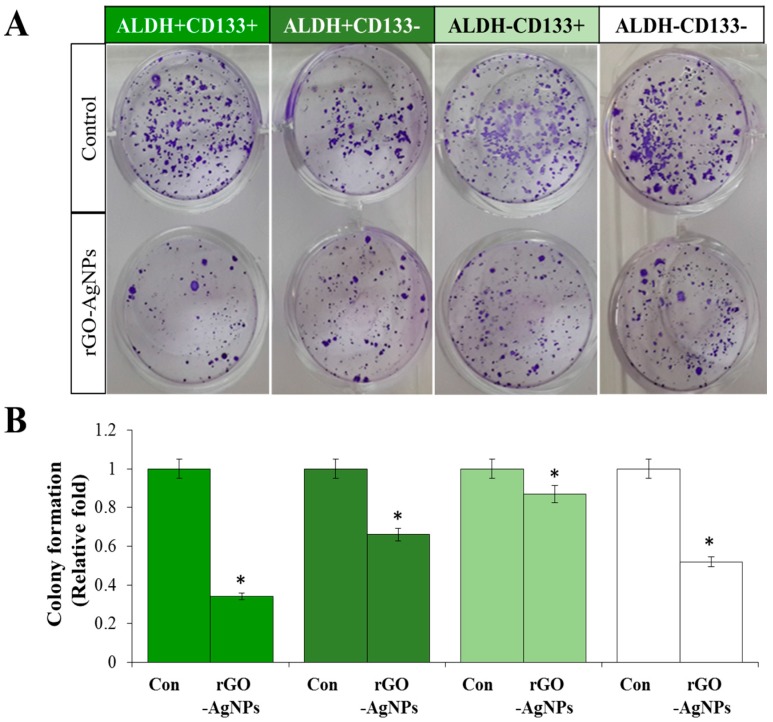
Effect of rGO–Ag on clonogenicity of various subpopulations of OvCSCs. Colony formation was quantitatively analyzed by crystal violet staining (**A**), the crystal violet was dissolved in methanol and then the absorbance was measured at 590 nm, (* *p* < 0.05) (**B**).

To corroborate the results of the cell viability assay, we performed a clonogenic assay. A clonogenic assay is considered more sensitive for evaluating toxicity than a cell viability assay, as colony formation is assessed when the cells are in a state of proliferation and are thus more susceptible to toxic effects [84]. Additionally, this method can be used to evaluate self-renewal and differentiation at the single-cell level. To confirm the anticancer effect of rGO–Ag, we performed a clonogenic assay in different subpopulations of OvCSCs including ALDH^+^CD133^+^, ALDH^+^CD133^−^, ALDH^−^CD133^+^, and ALDH^−^CD133^−^. The cells were seeded at the same density and incubated with 2 µg/mL rGO–Ag nanocomposite for 3 weeks, and colony formation ability in Matrigel was determined after 3 weeks. We cultured all four subpopulations of OvCSCs with the rGO–Ag for 3 weeks, and the numbers of colonies were calculated; the results showed that rGO–Ag significantly reduced colony formation in all four different subpopulations compared to the control. The rGO–Ag significantly reduced colony formation in all populations of cells (Figure 5A). Interestingly, the numbers of colonies were significantly reduced in the ALDH^+^CD133^+^ population of cells. Although we treated each subpopulation with equal concentrations of rGO–Ag, differential responses were observed in all populations tested, such as ALDH^+^CD133^+^, ALDH^+^CD133^−^, ALDH^−^CD133^+^, and ALDH^−^CD133^−^ cells showing losses of viability of 66%, 34%, 13%, and 48%, respectively (Figure 5B). The clonogenic assay indicated that among the four different subpopulations of OvCSCs, ALDH^+^CD133^+^ showed greatest sensitivity. The results obtained from the colony-forming assay are consistent with those of the cell viability assays. The loss of viability of all four subpopulations of cells showed differential responses to rGO–Ag.

It was previously reported that salinomycin significantly ruptured lung cancer tumorospheres from ALDH^+^ A549 lung cells in vitro [85]. Recently, anthothecol-encapsulated PLGA-nanoparticles exhibited an inhibitory effect against cell proliferation and colony formation and consequently induced apoptosis in pancreatic CSCs and cancer cell [86]. Nanoparticles can target important pathways such as the Wnt/β-catenin signaling pathway, notch, and transforming growth factor-β [87,88,89]. Recently, Choi et al. demonstrated that ALDH^+^CD133^+^ OvCSCs exhibited the greatest engraftment potential and generated tumors within 2–4 months, whereas ALDH^−^CD133^−^ cells from primary samples were unable to initiate tumors [15].

### 2.6. rGO–Ag Nanocomposite Causes Cytotoxicity and Loss of Mitochondrial Membrane Potential in OvCSCs

**Figure 6:**
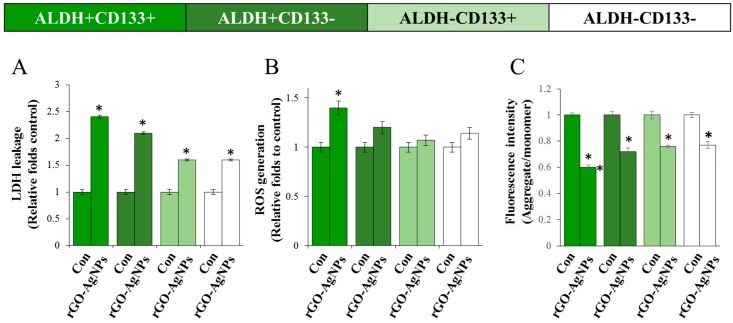
Effect of rGO–Ag on the leakage of LDH, reactive oxygen species (ROS) generation, and mitochondrial membrane potential (MMP) in OvCSCs. Different subpopulations of OvCSCs were incubated with rGO–Ag (2 µg/mL) for 24 h. (**A**) LDH activity was measured at 490 nm using the LDH cytotoxicity kit; (**B**) ROS generation was measured using 2′,7′-dichlorofluorescein; (**C**) Measurement of MMP in OvCSCs. The results are expressed as the mean ± standard deviation of three independent experiments. The treated groups showed statistically significant differences from the control group according to Student’s *t*-test (* *p* < 0.05).

Activation of cell death may also contribute to the toxicity of nanomaterials. Thus, nanoparticles can trigger either autophagy or apoptotic or necrotic cell death in primary cultures or cell lines [90]. To determine the mechanism of toxicity, several cellular enzyme assays are utilized, including lactate dehydrogenase (LDH), adenylate kinase, and glucose-6-phosphate dehydrogenase. Among them, only LDH is stable. Therefore, cell death assays based on LDH activity are more reliable than other enzyme-based cell death assays. Further, leakage of LDH is a well-known indicator of cell membrane integrity and cell viability [35]. LDH leakage results from the breakdown of the plasma membrane and alterations in membrane permeability, and is widely used as a cytotoxicity endpoint. This assay enables evaluation of cell death in cultures as a result of cell necrosis [91]. Cell toxicity was assessed by measurement of the amount of LDH leakage in the cell culture medium at 24 h in four different subpopulations of cells treated with rGO–Ag. All sub-populations of cells released LDH into the media (Figure 6A). Among the different subpopulations of cells, the ALDH^+^CD133^+^ subpopulation showed the greatest sensitivity. This indicates that ALDH^+^CD133^+^ subpopulations of OvCSCs are more sensitive than other populations of cells because of their suppressive activities against nanoparticles.

Next, we examined cytotoxic effects in an ROS generation assay. The results demonstrated that all four subpopulations, i.e., ALDH^+^CD133^+^, ALDH^−^CD133^+^, ALDH^+^CD133^−^, and ALDH^−^CD133^−^ produced ROS, but the values were low. ALDH^−^CD133^+^ and ALDH^−^CD133^−^ cells produced negligible amounts of ROS compared to ALDH^+^CD133^+^ (Figure 6B). Among the four different subpopulations, the effect of rGO–Ag on ROS production was significant in ALDH^+^CD133^+^. ROS in cancer cells were elevated partially because of their higher metabolism rates. ROS levels in cancer stem cells were lower because the drug-resistant or chemoresistant CSC population may use redox regulatory mechanisms to promote cell survival and tolerance to anticancer agents [92]. CSCs, similar to normal stem cells, are quiescent, slow-cycling cells with a lower level of intracellular ROS, accounting for their self-renewal capacity and resistance to chemotherapy drugs and ionizing radiation [93,94]. Further, Diehn et al. [93] showed that subsets of CSCs in tumors contained lower levels of ROS and enhanced ROS defenses compared to their non-tumorigenic progeny, contributing to radio-resistance. The possible reasons for the lower levels of ROS in CSCs are less ROS production and/or enhanced ROS scavenging systems; furthermore, the slow division of CSCs may generate less ROS than cancer cells [95]. The reason for the low levels of ROS in CSCs compared to bulk cancer cells may be because of the high antioxidant capacity to maintain cellular ROS at a moderate level and maintain both stemness and cancer-forming capabilities [93,96]. Our results are consistent with those of previous studies reporting lower levels of ROS in human gastrointestinal-derived stem-like populations (CD44 high) and CSCs from human and murine breast tumors [93,97].

Mitochondrial membrane potential (MMP) reflects the functional status of the mitochondrion related to cancer malignancy [98]. Recent studies suggested that mitochondrial features differ in CSCs with respect to MMP and ROS [93,96]. Mitochondria are the source of intracellular ROS. However, the link between ROS and MMP is unknown in CSCs. ROS mediated death was analyzed by the level of MMP and expression of pro- and anti-apoptotic genes [99,100]. There have been no reports of the effect of rGO–Ag in OvCSCs. Changes in MMP were analyzed using mitochondrial fluorescence dye, JC-1, which stains mitochondria in a membrane potential-dependent manner, in all four different subpopulations of cells treated with rGO–Ag. As shown in Figure 6C, cells exposed to 2 µg/mL rGO–Ag exhibited a significant decrease in the ratio of aggregate to monomer forms. Alterations in MMP cause apoptosis via depolarization of the mitochondrial membrane in bulk cells and a subpopulation of CSCs. Mitochondrial membrane potential (MMP) determines the functional status of mitochondria, including various cellular processes such as cell differentiation status, tumorigenicity, and malignancy [96] and also various apoptotic processes by the mechanism of release of apoptotic proteins, such as cytochrome c and second mitochondria-derived activator of caspase (Smac) [101,102]. The functional status of mitochondria depends on MMP, which is highly related to cancer malignancy. Mitochondrial permeability transition has been associated with various metabolic consequences, including inhibition of the electron transport chain with enhanced levels of ROS, and decreased production of ATP [103]. Previously, we reported that silver nanoparticles influence the MMP in various types of cancer cells such as human lung epithelial adenocarcinoma cells A549 [104] and human breast cancer cells [104,105]. Graphene inhibited electron transfer chain complexes I, II, III, and IV by disrupting the electron transfer between iron-sulfur centers [106]. These data suggest that rGO–Ag regulate the level of MMP and in turn induce apoptosis in CSCs.

### 2.7. Effect of rGO–Ag Nanocomposite on Expression of Pro- and Anti-Apoptotic Genes

**Figure 7:**
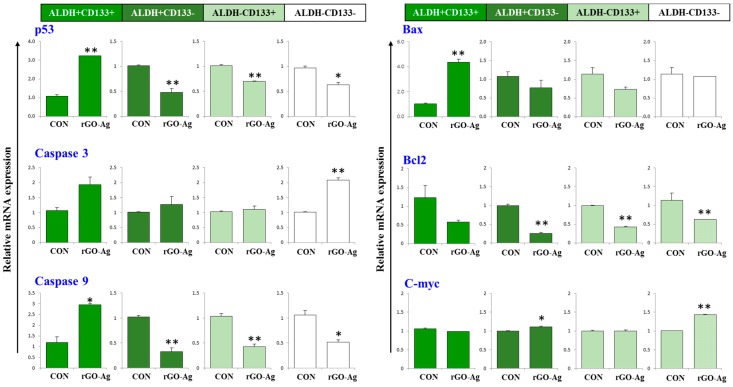
Impact of rGO–Ag on expression of apoptosis-regulated genes in OvCSCs. Relative mRNA expression of various apoptotic genes was analyzed by qRT-PCR in OvCSCs after treatment with rGO–Ag (2 µg/mL) for 24 h (**A**,**B**). The results are expressed as the mean ± standard deviation of three separate experiments. The treated groups showed statistically significant differences from the control group according to Student’s *t*-test (* *p* < 0.05, ** *p* < 0.01).

Apoptotic and anti-apoptotic genes play an important role in cell survival and apoptosis. We examined the effects of rGO–Ag on the expression of the *p53*, *caspase-3*, *caspase-9*, *Bax*, *Bcl-2*, and *c-myc* genes. OvCSCs were treated with rGO–Ag (2 µg/mL) and were incubated for 24 h. To corroborate the cytotoxicity caused by the rGO–Ag nanocomposite, the expression levels of some apoptosis-related genes, namely *p53*, *caspase-3*, *caspase-9*, *Bax*, *Bcl-2*, and *c-myc*, were determined by quantitative reverse transcriptase (qRT)-PCR (Figure 7A,B).

The process of apoptosis is positively regulated by tumor-suppressor p53, which induces the expression of many pro-apoptotic genes, including death receptors and multiple pro-apoptotic *Bcl-2* family members [107,108]. Activation of p53 leads to suppression of cell growth and induces apoptosis in ALDH^+^CD133^+^. Similarly, p53 suppresses proliferation and self-renewal of neural stem cells [109]. We examined whether apoptosis, triggered or sensitized by *c-myc*, is p53-dependent or p53-independent, and the results indicated that a high level of expression of *p53* induces apoptosis. The expression of *c-myc* depends on the specific cell type and physiological status of the cell [107]. Deregulation of *c-myc* causes apoptosis in the Rat-1 fibroblast cell line and primary rat embryo fibroblasts by either a thymidine block or isoleucine starvation [110]. Our findings suggest that downregulation of *c-myc* triggers apoptosis along with p53 (Figure 7A,B).

The Bcl-2 family proteins accelerate cell death by the mechanism of cytochrome c release and release of apoptogenic molecules from mitochondria to the cytosol and accelerate apoptotic cell death [111,112,113,114,115]. For instance, the imbalance level of pro and anti-apoptotic genes is responsible for mitochondrial dysfunction and energy depletion in CD34^+^ CML stem cells and ROS-low leukemia stem cells [116,117]. rGO–Ag clearly down-regulates *Bcl-2* and up-regulates *Bax* expression in the ALDH^+^CD133^+^ subpopulations of cells (Figure 7B). Similarly, berberine liposome induces apoptosis by down-regulating *Bcl-2* and up-regulating *Bax* in colon CSCs [118]. Similarly, we observed rGO–Ag causes imbalance in the level of Bcl-2 and Bcl-xl.

Caspases are known to be involved in apoptosis through two different pathways: intrinsic and extrinsic. The loss of mitochondrial membrane potential may promote activation of cytochrome c and mitochondria-derived caspases. The results of our experiment suggest that rGO–Ag up-regulates the expression of both caspase-9 and caspase-3 in ALDH^+^CD133^+^ subpopulations (Figure 7A). Our results agree with those of previous studies, demonstrating that natural compounds such as 20(*S*)-ginsenoside Rg_3_ inhibit the proliferation of colon CSCs and induce apoptosis through caspase-9 and caspase-3 pathways, and morusin induces apoptosis of cervical CSCs by down-regulating *NF-κB*/*p65* and *Bcl-2* and up-regulating *Bax* and *caspase-3* in a dose-dependent manner [119,120]. Previous results and our data suggest that apoptotic signaling pathways are significantly deregulated in ALDH^+^CD133^+^ subpopulations of CSCs. In contrast, other subpopulations of cells were not significantly impacted by the rGO–Ag. Overall, our results suggest that the rGO–Ag can activate apoptotic genes such as *p53*, *caspase-3*, *caspase-9*, *Bax*, and *c-myc* and can down-regulate *Bcl-2*. Thus, incubation of OvCSCs with rGO–Ag can sensitize ALDH^+^CD133^+^ by down-regulating anti-apoptotic *Bcl-2* and up-regulating pro-apoptotic gene expression such as *p53*, *caspase-9*, *caspase-3*, and *Bax*. Therefore, we selected ALDH^+^CD133^+^ subpopulations of cells to further evaluate the sensitivity of the combination of rGO–Ag and salinomycin.

### 2.8. Dose-Dependent Effect of rGO–Ag and Salinomycin on Cytotoxicity in ALDH^+^CD133^+^Cells

**Figure 8:**
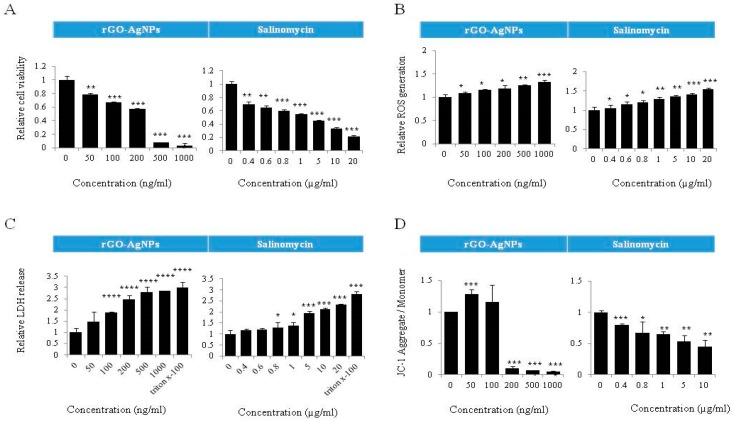
Dose-dependent effect of rGO–Ag and salinomcyin on cytotoxicity if ALDH^+^CD133^+^cells. ALDH^+^CD133+ cells were treated with various concentrations of rGO–Ag and salinomycin for 24 h. Cell viability was determined using a cell counting kit (CCK-8) assay (**A**); reactive oxygen species (ROS) generation was determined by 2′,7′-dichlorofluorescein diacetate (DCFDA) (**B**); lactate dehydrogenase (LDH) activity was measured at 490 nm using the LDH cytotoxicity kit (**C**); mitochondrial transmembrane potential (MTP) was determined using the cationic fluorescent indicator JC-1 (**D**). The results are expressed as the mean ± standard deviation of three independent experiments. The treated groups showed statistically significant differences from the control group according to Student’s *t*-test (* *p* < 0.05, ** *p* < 0.01, *** *p* < 0.005, **** *p* < 0.001).

To determine the sensitivity and optimize the dose for the combination effect by rGO–Ag and salinomycin on ALDH^+^CD133^+^cells, we first determined the dose response profile against the ALDH^+^CD133^+^ subpopulation of cells. ALDH^+^CD133^+^ cells were treated with various concentrations of rGO–Ag (50–1000 ng/mL) and salinomycin (0.4–20 µM) for 24 h. The results clearly indicated that both rGO–Ag and salinomycin had dose-dependent effects and that increasing concentrations of rGO–Ag or salinomycin strongly influenced cell viability, ROS generation, LDH leakage, and MMP loss (Figure 8). The cell viability results showed that the IC_50_ values of rGO–Ag and salinomycin were 200 ng/mL and 1 µM, respectively. Previous studies suggest that AgNPs have potential to induce apoptosis in A2780 cells and ALDH^+^CD133^+^ cells with an IC50 of 1000 ng/mL [121,122]. Interestingly, rGO–Ag and salinomycin may induce cytotoxicity at concentrations of 200 ng/mL and 1 µM because of the availability of both silver and reduced GO in the single platform and smaller size of AgNPs anchored on the surface of graphene sheets. Among the tested several cytotoxicity assays in ALDH^+^CD133^+^ cells, rGO–Ag induced a significant loss of MMP compared to leakage of LDH and ROS. Overall, the suppressive effect of rGO–Ag and salinomycin in ALDH^+^CD133^+^ cells was significant at low concentrations, indicating that subpopulations of ALDH^+^CD133^+^ are more sensitive to both rGO–Ag and salinomycin.

### 2.9. Combination Effect of rGO–Ag and Salinomycin on Cytotoxicity in ALDH^+^CD133^+^ Cells

**Figure 9:**
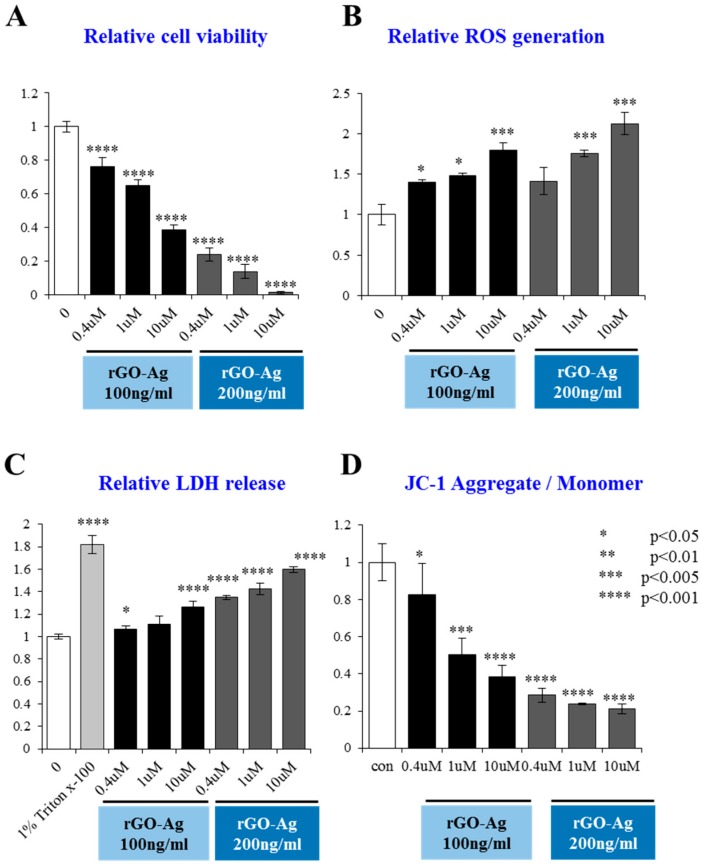
Effect of rGO–Ag, salinomycin and the combination of rGO–Ag and salinomycin on cytotoxicity. ALDH^+^CD133^+^ cells were treated with 100 ng/mL rGO–Ag plus 0.4, 1.0, and 10 µM of salinomycin and 200 ng/mL rGO–Ag plus 0.4, 1.0, and 10 µM of salinomycin for 24 h. Cell viability was determined using a cell counting kit (CCK-8) assay (**A**); reactive oxygen species (ROS) generation was determined by 2′,7′-dichlorofluorescein diacetate (DCFDA) (**B**); lactate dehydrogenase (LDH) activity was measured at 490 nm using the LDH cytotoxicity kit (**C**); mitochondrial transmembrane potential (MTP) was determined using the cationic fluorescent indicator JC-1 (**D**). The results are expressed as the mean ± standard deviation of three independent experiments. The treated groups showed statistically significant differences from the control group according to Student’s *t*-test (* *p* < 0.05).

**Figure 10:**
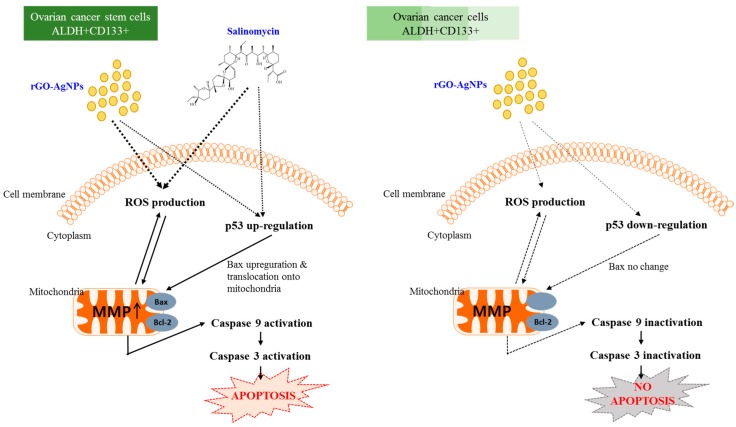
Hypothetical model explaining the working mechanism of rGO–Ag and salinomycin to induce toxicity and apoptosis in ovarian cancer cells and OvCSCs.

Salinomycin appears to selectively target cancer stem cells and eliminates both cancer stem cells and therapy-resistant cancer cells, indicating its potential as a novel and efficient chemotherapeutic drug [123]. Similarly, among metal nanoparticles, AgNPs appear to be potential therapeutic agents for CSCs and cancer cells [121]. No studies have reported the efficacy of rGO–Ag on CSCs, particularly ALDH^+^CD133^+^ cells. Hence, we selected two different molecules, rGO–Ag and salinomycin, to examine the combination effect on ALDH^+^CD133^+^ cells using a low concentration. ALDH^+^CD133^+^ cells were treated with two different combinations of rGO–Ag and salinomycin such as 100 ng/mL rGO–Ag plus 0.4, 1.0, and 10 µM of salinomycin as well as 200 mg/mL rGO–Ag plus 0.4, 1.0, and 10 µM of salinomycin for 24 h. Among the two different combinations evaluated in this study, both were toxic, and the second combination appeared to be highly cytotoxic in all tested assays including cell viability, ROS generation, LDH leakage, and MMP loss (Figure 9). The two different combinations caused severe mitochondrial dysfunction by inducing the loss of mitochondrial membrane potential, which is consistent with the dose-dependent toxicity of either rGO–Ag alone or salinomycin alone. These results also indicated that mitochondrial dysfunction is the primary source of ROS production and ultimately the increased level of ROS, leading to mitochondrial-mediated apoptosis and modulation of the down-regulation of anti-apoptotic *Bcl-2* and up-regulation of apoptotic gene expression in ALDH^+^CD133^+^ cells. Salinomycin was 100-fold more effective towards CSCs than the conventional chemotherapeutic drug paclitaxel and decreased the percentage of CD44^high^/CD24^low^ breast CSCs by 20-fold [124]. Hyaluronic acid-coated salinomycin nanoparticles decreased the expression of CD44 in breast CSCs and polysorbate 80-coated poly (lactic-co-glycolic acid)-encapsulated salinomycin nanoparticles enhanced cell death in glioblastoma [125,126]. The synergistic action of rGO–Ag and salinomycin induced apoptosis through caspase-dependent and caspase-independent pathways and was involved in the loss of membrane potential of mitochondria. The findings from this study revealed that the combination index shows CI < 1 and indicates synergy; our findings also support that the combination of rGO–Ag and salinomycin is suitable as alternative selective agents for killing CSCs and sensitizing tumor cells at a very low concentration. The hypothetical model revealed that rGO–AgNPs and salinomycin induce cytotoxicity and apoptosis in OvCSCs via oxidative stress (Figure 10).

## 3. Materials and Methods

### 3.1. Materials

Penicillin-streptomycin solution, trypsin-EDTA solution, Dulbecco’s modified Eagle’s medium, RPMI 1640 medium, and 1% antibiotic-antimycotic solution were obtained from Life Technologies/Gibco (Grand Island, NY, USA). AgNO_3_, fetal bovine serum, and the in vitro toxicology assay kit were purchased from Sigma-Aldrich (St. Louis, MO, USA). Graphite (Gt) powder, NaOH, KMnO_4_, NaNO_3_, anhydrous ethanol, 98% H_2_SO_4_, 36% HCl, 30% H_2_O_2_ aqueous solution, silver nitrate, R-phycoerythrin, and all other chemicals were purchased from Sigma-Aldrich unless otherwise stated.

### 3.2. Synthesis of AgNPs and GO

AgNPs were synthesized using R-phycoerythrin as described previously [35,46]. AgNPs were prepared by adding 1 mL of 4 µM RPE to 10 mL 5 mM aqueous AgNO_3_; the mixture was incubated for 6 h at 40 °C and pH 8.0. The bio-reduction of the silver ions was monitored spectrophotometrically at 420 nm. Further characterization of the synthesized AgNPs was performed as described previously [42]. GO was synthesized as described previously with suitable modifications [21,22,47].

### 3.3. Reduction of GO and Synthesis of rGO–Ag Nanocomposite by RPE

GO was reduced, as described previously [22,74]. GO, rGO, and rGO–Ag nanocomposites were characterized as described previously [21,22,74]. Briefly, rGO–Ag nanocomposites were prepared using RPE as a reducing and stabilizing agent. GO (100 mg) was mixed with 5 mM of AgNO_3_ in the presence of 10 mL of aqueous RPE (4 µM).

### 3.4. Flow Cytometry Analysis and Fluorescence-Activated Cell Sorting (FACS)

FACS was performed as described previously [13,15]. CSCs were sorted using CD133 primary antibodies and then examined for ALDH^+^ enzymatic activity using the ALDEFLUOR kit according to the manufacturer’s protocol (Stem Cell Technologies, Vancouver, BC, Canada).

### 3.5. Cell Viability, Measurement of LDH and ROS

The CCK-8 assay, cell membrane integrity and ROS measurement were performed as described previously [22,28,35,74] according to the manufacturer’s instructions.

### 3.6. Clonogenic Assay

The clonogenic assay was performed as described previously [121]. A2780 whole cells and sorted cells were plated into a 48-well plate at a density of 100 cells per well and allowed to adhere for 18 h. Concentrations of rGO–Ag (2 µg/mL) were added to each well and incubated for a maximum of 21 days at 37 °C. For each condition, three wells were used. Fourteen days after plating, the cells were washed and fixed with methanol, glacial acetic acid, and water (1:1:8 vol:vol:vol), and then stained with crystal violet. Colonies were counted and are expressed as plating efficiency relative to the control in the absence of rGO–Ag.

### 3.7. Mitochondrial Membrane Potential (MMP)

Mitochondrial membrane potential (MMP) was measured as per the manufacturer instructions (Molecular Probes, Eugene, OR, USA) and as described previously [22] using a cationic fluorescent indicator JC-1 (Molecular Probes).

### 3.8. Real-Time Quantitative Reverse Transcriptase Polymerase Chain Reaction (qRT-PCR)

Total RNA was extracted from cells treated with RPE–rGO–Ag using an Arcturus PicoPure RNA isolation kit (Thermo Scientific, Waltham, MA, USA) according to the manufacturer’s instructions. RNA was reverse-transcribed into cDNA using a Reverse Transcription Kit (Roche, Basel, Switzerland) in a final volume of 20 µL according to the manufacturer’s instructions. All gene transcripts (*p53*, *caspase-3*, *caspase-9*, *Bax*, *Bcl-2*, *c-myc*) were quantified in 3 replicates by real-time RT-qPCR on a Lightcycler apparatus using Lightcycler^®^ FastStart DNA Master SYBR Green I via an ABI Applied Biosystems machine (Foster City, CA, USA). The primer sequences for each gene are shown in Table 1.

### 3.9. Statistical Analyses

All assays were performed in triplicate, and each experiment was repeated at least three times. The results are presented as the means ± standard deviation. All experimental data were compared by Student’s *t*-test. A *p* value less than 0.05 was considered statistically significant.

## 4. Conclusions

CSCs have become a focus in cancer research. CSCs are a small population of cells that can self-renew and form tumors. CSCs are responsible for tumor recurrence, chemoresistance, drug resistance, and relapse of cancers and significantly affect tumor therapy. Thus, a CSC-focused therapy approach is vital in any effective anticancer therapeutic strategy. Eradication of CSCs is currently a major challenge in cancer therapy, which can be achieved by using nanomaterials that target CSCs. Here, we developed a simple, environmentally friendly, dependable, and non-toxic approach for synthesizing rGO–Ag using RPE. The cytotoxic potential of RPE-mediated synthesis of rGO–Ag was evaluated in ovarian cancer cells and different subpopulations of OvCSCs using various cellular assays. The results suggest that rGO–Ag is more cytotoxic than the other tested nanomaterials both in bulk cells (A2780) and the subpopulation of OvCSCs, exclusively to ALDH^+^CD133^+^ cells which are known to have high tumorigenic potential. To support the results of the biochemical and cellular assays, we performed a colony formation assay, which clearly showed that rGO–Ag significantly reduced the number of colonies. Furthermore, the mechanism of cytotoxicity was confirmed by various cytotoxicity assays, enhanced expression of pro-apoptotic genes, and down-regulation of the anti-apoptotic gene *Bcl-2*. The results indicate that rGO–Ag can be used to specifically target ALDH^+^CD133^+^ cells in a sensitive manner, providing a possible approach for cancer therapy with fewer side effects. This is the first study to demonstrate specific targeting of the ALDH^+^CD133^+^ subpopulation of CSCs by rGO–Ag. Further, the combination of rGO–Ag and salinomycin induced potential cytotoxicity in ALDH^+^CD133^+^ and efficiently targeted ALDH^+^CD133^+^ at a very low concentration. In addition, nanoparticle-mediated combination therapy may overcome induced mutagenesis and frequently relapsed tumors caused by chemotherapeutic agents. Clinically, a combination treatment involving nanoparticles and salinomycin that targets tumor-initiating cells may facilitate the removal of all cancer cells.

## Figures and Tables

**Table 1:** List of primers used in this study.

Gene	Primers
*Bcl-2*	F: ATGTGTGTGGAGAGCGTCAA
	R: GCCGGTTCAGGTACTCAG TC
*c-myc*	F: AGCGACTCTGAGGAGGAACA
	R: CTCTGACCTTTTGCCAGGAG
*p53*	F: TTTGGGTCTTTGAACCCTTG
	R: CCACAACAAAACACCAGTGC
*Bax*	F: ATGGAGCTGCAGAGGATGAT
	R: CAGTTGAAGTTGCCGTCAGA
*Caspase-3*	F: CATACTCCACAGCACCTGGTTA
	R: ACTCAAATTCTGTTGCCACCT T
*Caspase-9*	F: ACTTTCCCAGGTTTTGTTTCCT
	R: GAAATTAAAGCAACCAGGCATC
